# Correction to: Comparative phylogenetic analysis and transcriptomic profiling of dengue (DENV-3 genotype I) outbreak in 2021 in Bangladesh

**DOI:** 10.1186/s12985-023-02151-7

**Published:** 2023-08-18

**Authors:** Md. Murshed Hasan Sarkar, M. Shaminur Rahman, M. Rafiul, Arafat Rahman, Md. Shariful Islam, Tanjina Akhtar Banu, Shahina Akter, Barna Goswami, Iffat Jahan, Md. Ahashan Habib, Mohammad Mohi Uddin, Md. Zakaria Mia, Md. Ibrahim Miah, Md Aftab Ali Shaikh, Md. Salim Khan

**Affiliations:** 1 Bangladesh Council of Scientific and Industrial Research (BCSIR), Dhaka, Bangladesh; 2 Department of Microbiology, Jashore University of Science and Technology, Jashore, Bangladesh; 3 Department of Microbiology, University of Dhaka, Dhaka, Bangladesh; 4 Department of Microbiology, Noakhali Science and Technology University, Noakhali, Bangladesh; 5 Department of Microbiology, Jagannath University, Dhaka, Bangladesh

**Correction: Virology Journal (2023) 20:127**

10.1186/s12985-023-02030-1

Following publication of the original article [1], the authors informed us that the Abstract has been missed to be added.

The original article has been corrected.
