# Supplementary figures and images for: Comparative phylogenetic analysis and transcriptomic profiling of Dengue (DENV-3 genotype I) outbreak in 2021 in Bangladesh
Source: Virol J. 2023 Jun 19;20:127. doi: 10.1186/s12985-023-02030-1 (PMC10278332; doi:10.1186/s12985-023-02030-1)

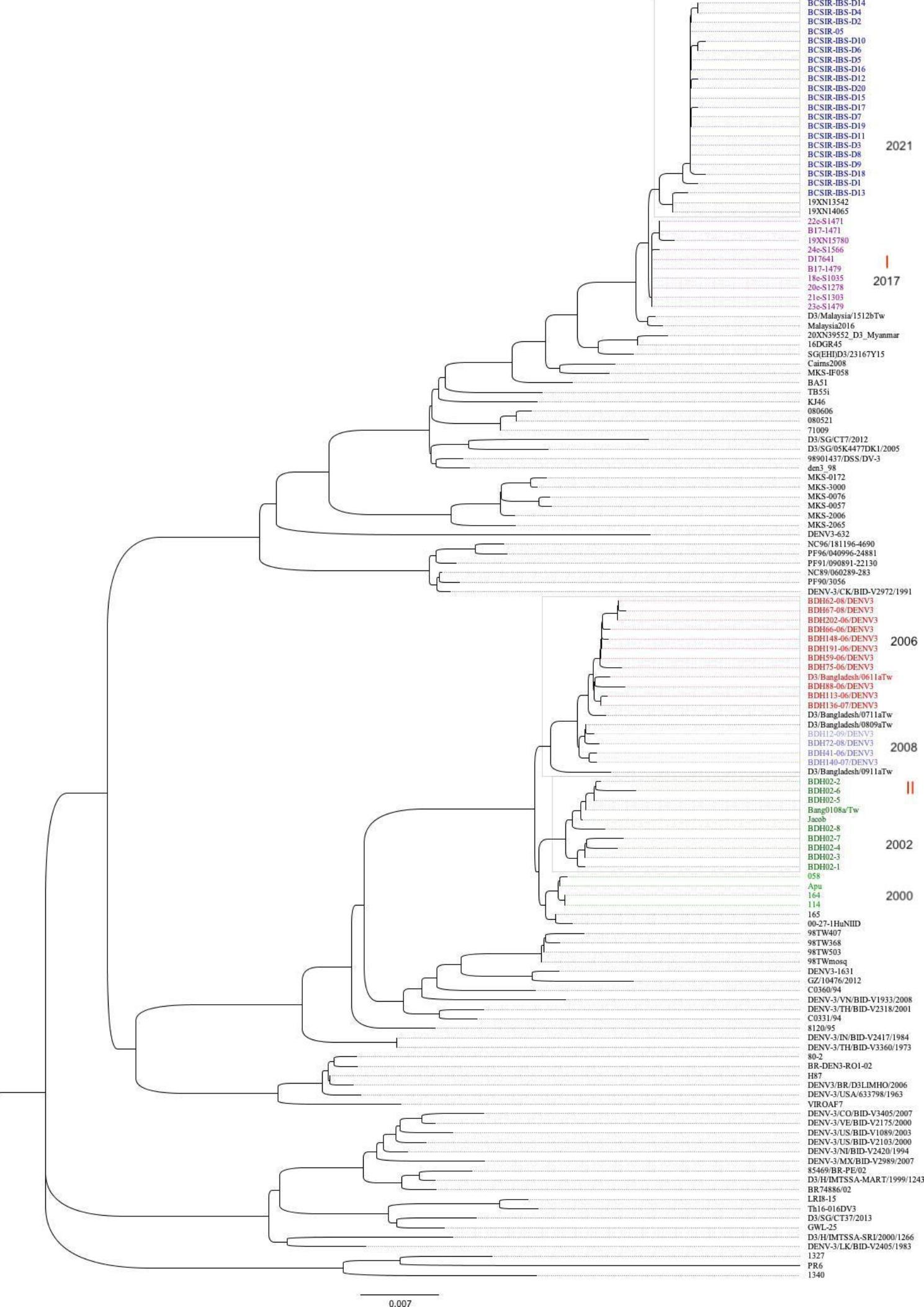

Supplement: Supplementary file 2 — Supplementary Material 2: Phylogeny of env gene highlighting DENV3 epidemics in Bangladesh. Isolates from all previously reported DENV3 in Bangladesh highlighted in contrasting color along with the year. The 2021 DENV3 epidemic is consistent with previously reported clad switch in 2017 from genotype II to genotype I [file 12985_2023_2030_MOESM2_ESM.png]
